# Michigan

**Published:** 1896-09

**Authors:** 


*Michigan.* A very fatal form of disease is reported among the sheep of St. Joseph County. It prevails as an enzoötic and is causing great losses.

